# Analysis of Occupational Therapy Students’ Pedagogical Practices for the Forging of Professional Identity and Development of Professional Intelligence: A Scoping Review

**DOI:** 10.3390/jintelligence11030048

**Published:** 2023-02-28

**Authors:** Ana-Isabel Souto-Gómez, Miguel-Ángel Talavera-Valverde, Luis-Javier Márquez-Álvarez, María-del-Pilar García-de-la-Torre

**Affiliations:** 1 Integra Saúde Unit Research, Escola Universitaria de Traballo Social, Universidade Santiago de Compostela, 15704 Santiago de Compostela, Spain; 2 Integra Saúde Unit Research, Health Science Department, Facultad de Ciencias de la Salud, Universidade da Coruña, 15570 A Coruña, Spain; 3 Área Sanitaria de Ferrol, 15405 Ferrol, Spain; 4 Área Sanitaria de Vigo, 36204 Vigo, Spain; 5 Psychology Department, Facultad de Ciencias de la Educación, Universidade da Coruña, 15008 A Coruña, Spain

**Keywords:** professional identity, students, occupational therapy, intelligent professional development, pedagogical practices, learning contexts

## Abstract

Pedagogical practices contribute to enhancing professional intelligence which is an indicator of maturity and development of professional identity. The research guiding question was: What are the pedagogical practices involved in occupational therapy students’ professional identity formation? A scoping review using a six-stage methodological framework was used to capture a variety of evidence describing how professional identity has been conceptualised and integrated into the occupational therapy curriculum while noticing a link to professional intelligence. Databases included were: Ovid MEDLINE, CINAHL, PsycINFO, ProQuest ERIC, Scopus, Web of Science, CSIC, Dialnet, PubMed, Pubmed Central, OTDBASE and Scielo. Qualitative content analysis was used to categorise learning outcomes into five components of professional identity that were associated with the pedagogical practices identified in the studies. *n* = 58 peer-reviewed journal articles were recorded. The articles were classified as intervention studies (*n* = 31; 53.4%), reviews (*n* = 12; 20.7%) and theoretical articles (*n* = 15; 25.9%). To ensure the feasibility of collecting and reporting results, we narrowed the focus to *n* = 31 intervention studies that provided information on pedagogical practices and learning outcomes on professional identity forging in students. This scoping review illustrates the variety of contexts in which students learn, the multiple dimensions of identity establishment, and the variety of pedagogical practices. These findings can be used to adapt and design focused formative curricula that support the development of professional identity.

## 1. Introduction

Professional identity refers to the attitudes, values, knowledge, beliefs, and skills shared within a professional group (Matthews et al. 2019). It is a multidimensional construct that evolves and changes over time (Walder et al. 2022). The process of forming professional identity involves acquiring knowledge, skills, and understanding of the reality and demands of the profession, as well as ethics, personal and professional values, and the moral context of practice (Dige 2009; Trede et al. 2012; Kahlke et al. 2020).

Professional identity is an individual’s professional self-concept based on beliefs, values, motives, and experiences (Healey and Hays 2012; Hurt-Avila and Castillo 2017). It exists in the context of a social and cultural role, and individuals often assume multiple identities and roles in their lives, including as a professional. Therefore, professional identity represents a set of traits that characterize individuals or communities within their professional context (Olesen 2001).

The development of professional identity is a longitudinal process that requires a well-delineated training. Students acquire the necessary skills, knowledge, and attitudes to become part of a professional group or discipline through a variety of variables (Matthews et al. 2019). The foundations for professional identity need to be established early in the process (Ashby et al. 2016).

Professional intelligence is characterized by a high degree of novelty and uniqueness (Sternberg 2020). It involves a person’s ability to perform their job effectively, understanding the methods and techniques necessary for success, such as problem-solving, decision-making, innovation, communication, and empathy (Sternberg 2020, 2021a). Adaptability and flexibility are also crucial skills for professional success (Sternberg 2021a; Sternberg et al. 2022a).

Professional intelligence is composed of three levels, which require specific skills that are acquired through training (Doublestein et al. 2020). The cognitive level includes focused thinking, problem-solving, critical thinking, decision-making, and explanation. The emotional level involves self-awareness, self-regulation, interpersonal relationships, empathy, and motivation. The leadership level requires embodiment, inspiration, empowerment, and recognition.

Professional identity and professional intelligence are interdependent and complementary aspects of a person’s professional life. The acquisition of professional skills from professional intelligence has a positive effect on the development of professional identity, and both are important for success and job satisfaction (Kushch 2019).

The close relationship between professional identity and professional intelligence suggests that the two concepts are mutually reinforcing. Professional identity relates to an individual’s perception of themselves in the context of their job or career (Walder et al. 2022), while professional intelligence refers to an individual’s ability to perform tasks and make effective decisions in a work environment (Sternberg 2021b; Sternberg et al. 2022b). The positive association between professional intelligence and professional identity indicates that a person with high professional intelligence is more likely to have a positive perception of their professional identity (Walder et al. 2022), and that a strong professional identity can enable individuals to have more confidence in developing their professional intelligence (Sternberg 2020, 2021a; Sternberg et al. 2022b). Therefore, it is essential to acknowledge the direct link between professional identity and professional intelligence, as both require the knowledge of professional and reasoning skills, academic training, and clinical practice to develop.

Occupational therapists require professional intelligence to adapt to the working environment and develop a common good within the discipline. The foundation on which professional intelligence develops is based on two elements: adaptive intelligence and practical intelligence (Sternberg 2020, 2021a; Sternberg et al. 2022a). The former refers to the skill of adapting to changing situations, while the latter involves using knowledge and experience to solve problems and make decisions. Professional intelligence can be considered a part of professional identity, as it encompasses interaction, intellectual, therapeutic, and management skills in a given task or situation.

Both professional identity and professional intelligence are multi-dimensional concepts that can be developed and strengthened by each other. The dimensions that make up professional identity, such as reputation, approach, values, commitment, and adaptation (Souto-Gómez et al. 2020), are matched by the development of the dimensions and skills that make up professional intelligence, including leadership, interpersonal, intrapersonal, and cognitive skills (Sternberg 2020, 2021a, 2021b; Sternberg et al. 2022a, 2022b). This generates a professional who has a good level of identity and is capable of adapting to various situations.

In the field of occupational therapy, the development of professional identity and professional intelligence is interconnected and essential to achieve a positive reputation among colleagues. Professional identity refers to understanding oneself as a professional, while professional intelligence involves effectively applying profession-specific knowledge, skills, and values (Mason 2006). Strong training curricula are necessary to develop both concepts throughout a therapist’s professional life (Ikiugu and Rosso 2003; Mason 2006). Educational support theories such as constructivism and problem-based learning promote student-centered teaching and practical application of knowledge, contributing to the development of professional intelligence, while identity formation theories such as Erikson’s (1968) psychosocial and identity development and those of Loevinger (1969, 1976) influence the understanding of how occupational therapy students develop their own professional identity over time. The aim of these theories is educational inclusion to guarantee equal learning opportunities for all students (Booth and Ainscow 2000). These theories of teaching support provide the necessary tools for learning, so that each student can develop their own autonomy with respect to everyday life situations, both in those related to training and professional practice (Booth and Ainscow 2000).

However, occupational therapy training has its peculiarities and requires a tailored curriculum that prepares students for their responsibilities towards society, clients, colleagues, and themselves. Universities must nurture and support students’ professional identity to strengthen it (Ventres et al. 2018) by developing skills like resilience, well-being, and meeting the requirements of professional and ethical practice (Brown et al. 2019) to prepare them for their working activity (Trede et al. 2012).

However, there is a lack of consensus on definitions and relationships between terms in occupational therapy professional discourse, such as paradigms, models, theories, and frames of reference, which hinders the development of a solid occupational therapy identity. Without a clear and specific terminology and taxonomy, shaping a professional identity will continue to be a challenge (Walder et al. 2022).

Two key points draw our attention. Firstly, research on professional intelligence and professional identity has been extensively conducted in areas such as military intelligence (Dylan et al. 2017), logistics management (Yang and He 2017), psychology (Mandel et al. 2022), marketing (Stastny and Lehner 2018), and computer science (Scholtz 2011). However, there is no study in occupational therapy that examines the relationship between both concepts. As a result, it is crucial to comprehend the pedagogical practices that foster the development of professional identity as they directly affect the components of professional intelligence and, consequently, enhance it.

Secondly, studies on pedagogical practices are imperative to gain a comprehensive understanding of the development of professional identity. Although such research exists in other fields such as nursing (Simmonds et al. 2020), pharmacy (Noble et al. 2019), and medicine (Wald et al. 2015), there is no specific taxonomy for these practices in occupational therapy, despite the efforts made by the WFOT (2016). Therefore, it is essential to explore the relationship between personal and professional values, structural and power influences, discipline versus generic education, the role of workplace learning in professional identities, and recommended pedagogical practices for professional identity development (Trede et al. 2012).

This study aims to (a) describe the nature and extent of research conducted on professional identity in occupational therapy, (b) identify pedagogical practices or learning contexts that contribute to professional identity formation in occupational therapy education, linked to the development of professional intelligence, and (c) map the components of professional identity described within these practices.

## 2. Materials and Methods

A scoping review was conducted following the PRISMA-SCR guidelines (Tricco et al. 2018), using the methodological framework established by Arksey and O’Malley (2005) and further developed by Levac et al. (2010). This method will help clarify a concept as complex as professional identity formation, in addition to capturing and mapping a variety of evidence to illustrate the scope of the study area. The protocol was registered in the Open Science Framework (retrospective registration).

### 2.1. Identification of the Research Question

The research question used was: How are the pedagogical practices involved in the formation of the professional identity of occupational therapy students?

### 2.2. Identification of Relevant Studies

The research team included occupational therapy professors with methodological, theoretical, and practical experience, as well as a health sciences documentalist, curriculum consultant, and research assistant. 

The initial search strategy began by writing a definition of professional identity formation, from which search terms were developed and synonyms were identified, (using examples, related ideas and synonyms). The synonyms identified were (a) from the discipline: baccalaureate occupational therapy education, educational therapy undergraduates, occupational therapy schools, occupational therapy students; (b) from the identity: social identification, social identity, professional socialization, self-concept role, occupational therapy role, professional identity, identity formation and self-perception.

The following databases were consulted on 9 October 2021: Ovid MEDLINE, PsycINFO, ProQuest ERIC, Scopus, Web of Science, CSIC, Dialnet, PubMed, Pubmed Central, OTDBASE, and Scielo. Search strategies were adjusted by applying the descriptors to each database. We used headings from CINAHL, ASSIA, APA and ERIC Thesaurus, MeSH terms and keywords. Also, we identified additional studies by reviewing references of relevant articles. Using the same search criteria, and with the aim to include possible new articles, the search was conducted again on 30 December 2021.

The search strategy was adapted to the different databases. For PubMed the search strategy was: (“Baccalaureate occupational therapy education” OR “Occupational therapy undergraduates” OR “Occupational therapy schools” OR “Occupational therapy students”) AND (“Social identification” OR “Social identity” OR “Professional Socialisation” OR “Self-concept Role” OR “Occupational therapy role” OR “Professional identity” OR “Identity formation” OR “Self-perception”). The results were exported to the Zotero bibliographic reference manager (5.0.82). Duplicates were removed and the filtered results were transferred to Excel (V.16.16.16) to organize and manage the initial search results. Only peer review articles were included to ensure feasibility. 

### 2.3. Selection of Studies

#### 2.3.1. Inclusion Criteria

Peer review articles published in English, Spanish and Portuguese that addressed occupational therapy training (undergraduates, masters, doctoral level or baccalaureate) and described or recommended pedagogical practices and learning outcomes related to professional identity formation. Pedagogical practices had to encompass specific didactic strategies developed and implemented within occupational therapy programs to promote professional identity formation. There were no restrictions on publication date.

#### 2.3.2. Exclusion Criteria 

Conferences, theses, and opinion articles were excluded. Additionally, empirical studies presenting the perspectives of occupational therapy students on professional identity formation were excluded if they did not recommend pedagogical practices or provide results on professional identity formation.

### 2.4. Data Processing, Classification and Analysis

After selecting the full-text articles included in the study, the data table was designed and variables were defined. The table was updated twice after the appearance of variables initially not considered (Professional Identity Formation and Professional Identity Formation Learning) (Table 1). Two co-authors independently recorded seven studies to ensure a consistent approach and to ensure that the variable form collected sufficient data to answer the research question. 

Two co-authors (AISG and MATV), who compiled and reviewed studies (title, abstract and full- text), also conducted the mapping with Covidence Software to screen the articles and carry out the full-text review process, and subsequently discussed it with the other authors (MPGDT and LJMA). There were no article to debate after the review of titles, abstracts, and full-texts. 

Professional identity learning outcomes were categorised after theoretical saturation (Hernández-Sampieri and Torres 2018) into five components and linked to pedagogical practices and learning contexts identified in the studies. The quantitative variables were expressed using frequency and percentage. 

This study used abductive reasoning which combines deductive and inductive reasoning, adapting the theoretical framework to the empirical results. At the same time, these changes in the theoretical framework allow for a conceptual interpretation of the data. This type of reasoning is common when dealing with under-researched topics (Verd and Lozares 2016).

Using the qualitative content analysis approach suggested by Levac et al. (2010), and in the spirit of (a) validating and improving our understanding of our findings, (b) seeking feedback on the coherence and direction of our thematic analysis, and (c) serving as a knowledge transfer mechanism on the relevance of professional identity in occupational therapy training, we conducted two consultations (with approximately six months difference): (a) with experts (five lecturers and practice educators of two Spanish universities and five professionals working in mental health, paediatrics, community, physical rehabilitation and geriatrics) and (b) students (ten final year undergraduates from two Spanish universities and ten final year master students from two Spanish universities) of occupational therapy. They were recruited through a snowball sampling technique.

We presented the contents in a blinded way (no professional or student knew that others were being consulted). The first consultation was carried out at the beginning of the analysis with the first emerging data and categories. The second was at the end of the data analysis.

We have used JBI Levels of Evidence developed by the Levels of Evidence and Grades of Recommendation Working Group of the Joanna Briggs Institute (JBI 2014) to evaluate the evidence (Effectiveness and Meaningfulness) of the empirical intervention studies that described associations between pedagogical practices and professional identity formation outcomes for students.

## 3. Results

**Figure 1:**
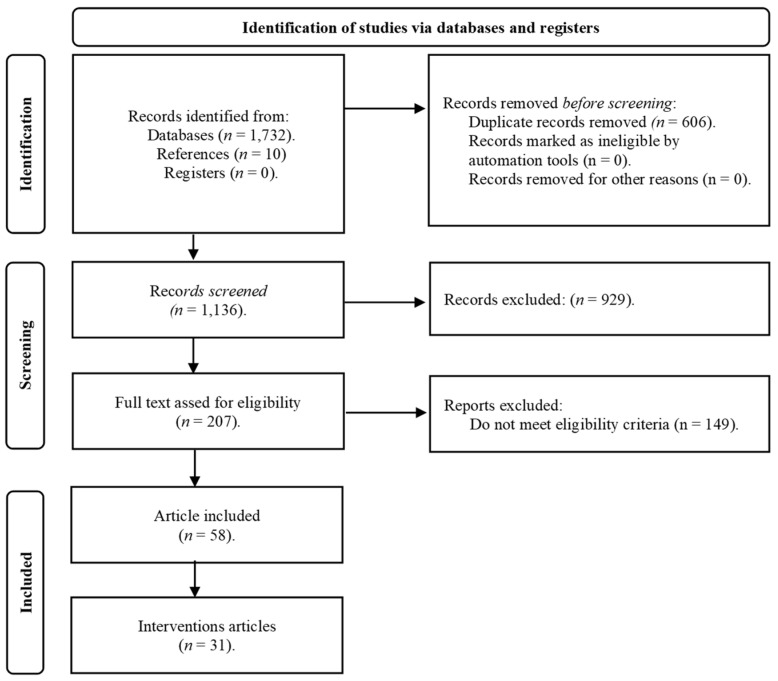
PRISMA Flowchart of record identification and study selection. Page et al. (2021).

A total of 1732 articles were identified, and after eliminating the duplicates and applying the eligibility criteria, 58 articles were selected for analysis (Figure 1).

### 3.1. Nature and Volume of Publication

Next, we will describe the volume and nature of research conducted on professional identity in occupational therapy.

The 58 peer-review articles were written between 1978 and 2021 in 11 different countries: the USA (28.8%), Australia (22%), the UK (18.6%), Canada (8.5%), Israel (6.8%), South Africa (3.4%) and Brazil, Colombia and Spain (1.7%), respectively. The remaining countries do not reach 1%.

One hundred and sixty-eight authors from 73 centres wrote the 58 articles. Sixty-two (84.9%) of these centres were universities, the remaining were hospitals and scientific societies. The universities of Queensland (Australia), McGill (Canada) and Monash (Australia) were the most active while the most prolific authors were Thomas, Y. and Ashby, S. The articles were grouped into three categories: (a) empirical research studies 31 (53.4%) which described specific pedagogical practices, concepts of professional identity formation, and recommendations for pedagogical practices for professional identity formation; (b) reviews: narrative (7; 12.1%), systematic (4; 6.9%), and scoping reviews (1; 1.7%) that provided content on professional identity formation and recommendations for pedagogical practices based on findings; (c) theoretical articles 15 (25.9%) with recommendations for pedagogical practices that address the formation of professional identity. 

#### Empirical Research

To ensure the feasibility of collection and reporting results, and to respond to the objective of identifying pedagogical practices or learning contexts that contribute to the formation of professional identity in occupational therapy education linked to the development of professional intelligence, we narrowed the focus to the 31 empirical intervention studies that described associations between pedagogical practices and professional identity formation outcomes for students.

From 1978 to 2010 the research was based on 6 (19.4%) qualitative studies, 1 (3.2%) quantitative studies and 2 (6.4%) mixed studies. The highest concentration of articles takes place in the decade of 2011–2021, amounting to 22 (71%). The frequency of empirical research studies increased in that decade, and the percentage of qualitative articles (11; 35.4%) exceeded the others, being the majority phenomenological ones (7; 22.6%). 

The sample of the 31 studies amounts to 3430 participants. Undergraduates were the most numerous with *n* = 2728 (79.5%). In the distribution of courses, the most studied were the first-year undergraduates, 19 (61.3%). There were 12 (38.7%) articles that did not incorporate information about the sample. As for gender, 11 (35.5%) articles did not provide information, and in the remaining 20 (64.5%), there were 1728 women and 358 men. Only one article described student’s ethnicity.

### 3.2. Pedagogical Practices or Learning Contexts That Contribute to the Forging of Professional Identity in Occupational Therapy Education Linked to the Development of Professional Intelligence

The 31 intervention studies investigated pedagogical practices in a variety of settings (classrooms, simulation laboratories, clinical practice settings or discussion groups). Some practices implied learning in multiple settings, for example, blending clinical experiences with classroom theory.

After reading the 31 intervention studies investigated on pedagogical practices and then categorising the data using abductive reasoning, we can say that five categories emerged (Table 2). Fieldwork (29%) and Reflexive practice (22.6%) are the most predominant learning contexts in the development of professional intelligence. 

All studies were at the evidence level (JBI 2014) four for effectiveness (Observational-Descriptive Studies), except Tal-Saban and Weintraub (2019) which was an Observational—Analytic Designs (Observational study without a control group). All studies were at level three for meaningfulness (JBI 2014), they were single qualitative studies. (Table 3).

### 3.3. Professional Identity Formation Components and Learning Outcomes

The concept of professional identity formation was described using different terms: professional socialisation, transition to the role, preparation for the role, professional preparation or acquisition of self-concept. Professional intelligence was described using the elements: compassion, agility, honesty, creativity, harmony, learning attitude, art of communication, personality, or persistence.

In order to respond to the objective, map the components of professional identity described within these practices, we have proceeded to describe the results obtained from the pedagogical practices.

**Figure 2:**
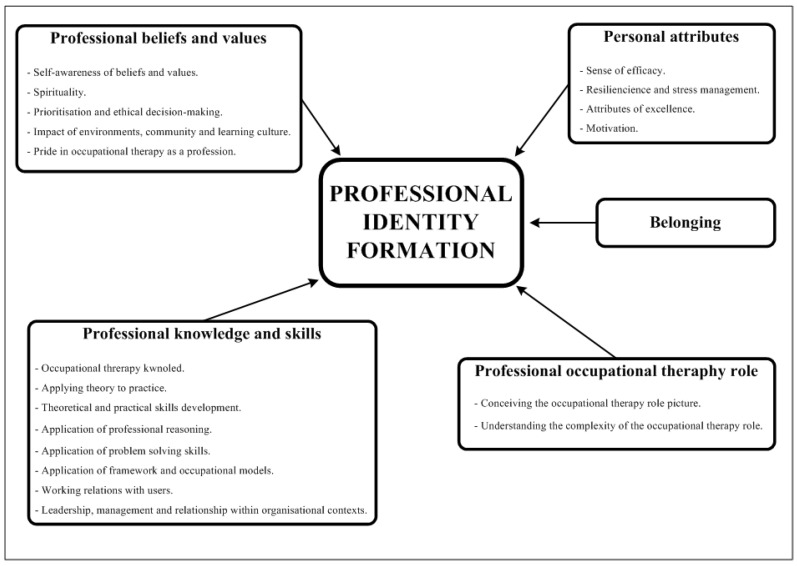
Components of professional identity formation and associated learning outcomes.

The pedagogical practices had multiple learning outcomes related to professional identity formation and professional intelligence. They were classified into five components: (a) professional knowledge and skills (14 studies), (b) professional beliefs and values (three studies), (c) personal attributes (five studies), (d) belonging and (e) understanding the occupational therapy role (nine studies) (Figure 2).

#### 3.3.1. Occupational Therapy Professional Knowledge and Skills

This was the most frequent component of professional identity development and professional intelligence in a variety of pedagogical practice settings.

We thought that classroom-based pedagogical practices, simulations, problem-based learning (PBL) and role-emerging placements (REP) were associated with professional identity learning outcomes and they, therefore, increase professional intelligence. These practices contributed to discipline knowledge, theoretical and practical skill development, leadership, management and the application of theory to practice.

Within the learning environments, REP (Golos and Tekuzener 2019; Thew et al. 2018), simulation and mentoring were quality practices and they supported the development of professional identity and professional intelligence (Walls et al. 2019).

Pedagogical practices that linked clinical and classroom contexts increased knowledge of practice models (Ashby et al. 2016), and the development of theoretical and practical skills (Humbert et al. 2012; Neistadt 1987) strengthened students’ relational work with users (Davis 2006), including the ability to function in organizational contexts (Rodger et al. 2014). 

Interventions with clients where students had to interact and make decisions to solve problems, Knecht-Sabres (2013), were associated with the development of professional interaction, technical, therapeutic, intellectual, organisational or managerial skills (Rodger et al. 2014; Talavera-Valverde 2015), favouring the increase of knowledge and professional skills (Humbert et al. 2012; Talavera-Valverde 2015) in the professional intelligence.

#### 3.3.2. Professional Beliefs and Values

Teaching environments were associated with learning outcomes through the understanding of the occupational therapy professional role (Greene 1997; Hammel et al. 1999). The development of professional beliefs and values, professional expectations and responsibilities were associated with classroom learning (Thompson and MacNeil 2006). In addition, learning outcomes of self-awareness of beliefs, values, self-reflection (Humbert et al. 2012; Brown et al. 2018), ethical decision-making, knowledge of the patient as a person (Walls et al. 2019), and reflection on complex ethical dilemmas (Neistadt 1987; Thompson and MacNeil 2006) were improved.

Beliefs and spirituality mark the understanding of what it means to be an occupational therapist (Thompson and MacNeil 2006). Classroom and clinical teaching strategies that integrated reflective components were associated with students’ motivation, increased self-awareness of their beliefs, and sense of belonging (Botkin 1979; Hammel et al. 1999; Psychouli et al. 2020). 

#### 3.3.3. Professional Occupational Therapy Role

The understanding of the professional role and the relationship with other roles determines the knowledge of the role of the occupational therapist within the work teams. Pedagogical practices in the classroom such as seminars (Thompson and MacNeil 2006), reflective discussion groups (Hammel et al. 1999; Parra-Esquivel et al. 2017; Psychouli et al. 2020), working groups for the use of different reasoning (Greene 1997; Neistadt 1987) and PBL (Barnard-Ashton et al. 2017; Stern 1997; Whitcombe 2013), were associated with learning outcomes related to the perceived role complexity of teams within working environments (Fitzgerald et al. 2017; Thew et al. 2018).

#### 3.3.4. Personal Attributes 

Learning outcomes associated with the development of confidence, self-efficacy, self-concept, and resilience were related to simulated and practice learning environments (Brown et al. 2019; Tal-Saban and Weintraub 2019). These results were linked to PBL pedagogical practices (Hammel et al. 1999; Whitcombe 2013), PBL skill development (Botkin 1979; Knecht-Sabres 2013), simulation (Haracz et al. 2015; Imms et al. 2018; Walls et al. 2019) and feedback to reinforce learning reasoning (Neistadt 1987).

Strategies in the classroom promoted confidence and self-efficacy, facilitating reflection on the image of occupational therapy (Thompson and MacNeil 2006; Walls et al. 2019). Experiences in the clinic developed motivation and inspired feelings of efficiency and stress coping (Brown et al. 2019). 

#### 3.3.5. Belonging

Students’ sense of belonging to the occupational therapy profession was developed in pedagogical practices designed for this purpose. These included REP (Golos and Tekuzener 2019; Knightbridge 2014; Thew et al. 2018) and knowledge consolidation under supervision in fieldwork (Davis 2006; Rodger et al. 2014; Hills et al. 2016; Tal-Saban and Weintraub 2019). 

Classroom-based strategies to heighten the sense of belonging included discussion groups (Botkin 1979; Hammel et al. 1999; Psychouli et al. 2020) and Brookfield’s model (Barnard-Ashton et al. 2017) which was also used by other disciplines but applied in this case to occupational therapy.

## 4. Discussion

The obtained results allowed us to achieve the research purposes. We wish to highlight three findings:

### 4.1. Volume and Nature

The number of field studies detected in relation to the volume was greater than that of reviews, as seen in the study by Snell et al. (2020), which leads us to believe that researchers have adopted a scientific approach in their studies of professional identity formation, thereby allowing them to control external factors that could affect the results and analyse the relationship between variables more precisely (Moruno-Miralles 2017).

In relation to the volume, a greater number of field studies was detected compared to reviews, the same as in the study by Snell et al. (2020), which makes us think about the high degree of experimentality of the studies on the formation of professional identity in occupational therapists (Moruno-Miralles 2017).

Publications from the USA and the UK predominate, as in the study by Van Schalkwyk et al. (2019). Australia and to a lesser extent, Canada and Israel are included, similar to the study by Snell et al. (2020). We detected a scarce contribution of these educational experiences in Spanish-speaking countries, which leads us to consider that this concept has not been sufficiently studied, and probably limits its understanding and development in certain contexts.

Our results point to two historical periods. The first one, exploration (1978–2010), characterised by empirical qualitative studies based on phenomenological approaches, indicating an exploratory perspective.

The second is a transition period (2011–2021), where the number of studies grows and diversifies. Quantitative studies appear related to the need to obtain results that can be generalized, as already highlighted by Ottenbacher and Petersen (1985).

We can sense an interest in this area of knowledge from 2011 to the present. We do not know if this growth is sufficient to speak of the arrival of a period of consolidation. We believe that the consolidation period would imply a collective process of positive identity configurations for the discipline, as it would allow the development of the concept, analysing the causes and consequences of the problem related to its understanding, turning occupational therapy into a mature discipline (Turner and Knight 2015).

As for the nature of the publications, they are focused on university work teams. This situation leads us to consider the importance of these groups to understand the characteristics that make up professional identity, the contexts and teaching approaches as pointed out by Boehm et al. (2015) and Mason (2006).

Furthermore, it should be noted that research on professional identity in occupational therapy is empirical and qualitative in nature, similar to the study by Snell et al. (2020). We believe this may be related to the predominance of qualitative research in occupational therapy during the 80s and 90s (Frank and Polkinghorne 2010), as it is the most appropriate for exploring the nature of professional identity due to the picture obtained from the perspective of the agents involved (Frank and Polkinghorne 2010; Walder et al. 2022).

Findings from this scoping review may be limited in several ways. Our findings were based on English language empirical studies published in peer-reviewed publications only. However, there may be important resources in other languages and grey literature. It is also possible that relevant studies were missed during our search process. Finally, although consistent with the goals and guidelines for scoping reviews, quality of research was not critically appraised. However, levels of evidence according to the Joanna Briggs Institute Approach (JBI 2014) were noted for all studies. The quantitative effectiveness evidence levels were three for the Observational—Analytic Designs and four for the Observational–Descriptive Studies of case studies; the qualitative meaningfulness level was at level three for qualitative studies or qualitative aspects of mixed-method designs.

### 4.2. Pedagogical Practices

While mapping the research learning environments, we highlight that pedagogical practices that foster professional identity are present in various contexts as noted by Adams et al. (2006) or Ikiugu and Rosso (2003). In the current review, we identified five, the first two being the most numerous: fieldwork, reflective practice, REP, simulation and others. This leads us to understand that professional intelligence linked to professional identity exists when the occupational therapist interacts with the task and situation (Sternberg 2021b).

We believe that research on professional identity in occupational therapy is concerned with the particularities of how identity is shaped through practical activities as opposed to lecturers or dissertations (Moruno-Miralles 2017). It is there where we appreciate the practical nature of the discipline, where teaching fosters the positive professionalism of identity (Moruno-Miralles 2017).

The transformation toward practice teaching environments as a learning context facilitates an understanding of professional identity (Ashby et al. 2016). The formation of this identity requires interpersonal, professional, judgmental, reasoning, critical thinking, and understanding of responsibilities skills (Cruess et al. 2019; Trede et al. 2012; Yu et al. 2018). The development of professional intelligence unrelated to professional identity is also linked to personal intelligence, when occupational therapists apply concrete personality patterns/models to set long-term goals and plans, according to the study conducted by Allen and Mayer (2022)

Concerning the two most numerous pedagogical practices in this study, we believe that fieldwork is the bulwark of the discipline as highlighted by Adams et al. (2006). Professional identity is constructed through an evolving and interactive process, which facilitates the student to develop a sense of professional “self” (Snell et al. 2020). Therefore, students with these types of fieldwork develop a stronger professional identity than students without them.

Fieldwork facilitates an early introduction to practice, as the earlier students have the opportunity to reflect on and articulate their experiences, the more beneficial it is for developing their professional identity (Snell et al. 2020). In this learning context, students continually adapt to the expectations of the role, constructing their identities, integrating experiences and developing professional skills (Adams et al. 2006; Benner 2011). In this context, supervisors or educators are responsible for accompanying and supporting by giving feedback to students to foster knowledge of the discipline and thus develop their professional identity (De Beer and Martensson 2015).

As for reflective practices, they are fundamental to transforming concepts and understanding them. Conscious and critical reflection (Schön 1984) allows learners to actively engage in the exploration of knowledge and experience, connecting theory and practice (Ikiugu and Rosso 2003). Pedagogical practices that link learning in different contexts and use conscious reflection help to address the dissonance of academic and clinical learning environments. They also support professional reasoning and the development of PBL, synthesis and evaluation skills needed to express ideas and improve professional performance (Talavera-Valverde 2015). 

Surprisingly to us, as happens with other disciplines such as nursing (Cruess et al. 2019), the complex and changing environments faced by occupational therapy students are useful in developing their identity as they facilitate a strong professional role by operationalising professional knowledge and skills (Fitzgerald et al. 2017; Yu et al. 2018). 

### 4.3. Components That Make Up the Professional Identity

The research consulted highlights the importance of the grade point average, learning styles, supervisor types, professional skills development and academic integrity as contributing factors in practice education performance (Brown et al. 2019; Walder et al. 2022; Yu et al. 2018). 

Learning is essential in students’ transition (Commission on Education, American Occupational Therapy Association 2009), and our results indicate that the development of occupational therapy professional knowledge and skills were the most addressed components of professional identity forging. This shows a discipline that emphasises the process of learning a role to create professionals with strong identities (Benner 2011; Davis 2006) with skills to cope with different environments that could arise (Sternberg 2021b). Adaptative intelligence changes according to the time and place and it is part of professional intelligence contributing to the development of resolution skills. 

In this regard, the teaching of professional beliefs and values is key for students to understand what it means to be an occupational therapist. According to Barbarà-i-Molinero et al. (2017) or Walder et al. (2022), commitment and personal development are related to the acquisition of professional identity. Concerning this, students engage in a process of socialization for work (acquisition of professional values and skills) Yu et al. (2018). Also, reflection on the role, professional practice, values, beliefs, ethical decisions, spirituality or what the profession means are key to understanding professional identity and feeling part of the discipline (Barbarà-i-Molinero et al. 2017; Peloquin 2008). 

The perception of being good at something or having an interest helps students to develop their professional identity (Hallier and Summers 2011). The teaching of these aspects facilitates decision-making, and the ability to resolve dilemmas that may arise and reinforces the development of other aspects that make up the professional identity (expectations, experiences or characteristics of the degree) Barbarà-i-Molinero et al. (2017), as it happens with cultural intelligence and crystalised intelligence. These two parts of professional intelligence stem from experience and contribute to understanding what has been learnt from an experience and how it helps to develop their professional identity and intelligence (Sternberg et al. 2022a, 2022b).

Continuing along these lines, the professional occupational therapy role is built on reflection. This reflection needs a theory that helps the students to reason professionally. The teaching of professional reasoning at a theoretical and practical level as a pedagogical activity facilitates the understanding of the role of an occupational therapist (Holmes et al. 2010; ENOTHE 2004; Walder et al. 2022). Recommendations to increase the number of hours of teaching this type of content are the exponent that professional reasoning and reflective practice facilitate the understanding of the identity of a profession (Holmes et al. 2010).

In addition, the personal skills that students present will influence the development of their professional identity. Therefore, in order to avoid an increase in the degree of burnout or the appearance of situations of low satisfaction and stress during their first year of practice (Fortune 2000; Mackenzie 2002; Tryssenaar and Perkins 2001), learning in fieldwork and simulation contexts is recommended, in which supervisors and lecturers and practice educators favour safe and inclusive learning environments where resilience is increased (Brown et al. 2019; Walder et al. 2022), anxiety is reduced, confidence is promoted and self-concept is developed (Roter 2006). 

Finally, the sense of belonging endows identity (Ennals et al. 2016). The sense of belonging is an important predictor of moral commitment and professional identity (Lindemann 2001). Being accepted, included, valued and encouraged, enables students to participate in relationships that shape their professional lives, enhancing competence and sense of self (Ennals et al. 2016; Walder et al. 2022).

Given the purpose of this research, we believe that this scoping review illustrates a multifaceted professional identity, a range of contexts in which occupational therapy students learn, as well as the breadth of pedagogical practices and learning outcomes that can guide the design of occupational therapy professional identity educational and development courses linked to the development of professional intelligence.

The results of this scoping review demonstrate that knowledge is slowly being produced about pedagogical practices or learning contexts that contribute to the formation of professional identity in Occupational Therapy education related to the development of professional intelligence in our profession. Nevertheless, the knowledge from the 58 articles located, although not overwhelming, stands out for being empirical (31 empirical articles versus 27 non-empirical). Perhaps the low quality of the studies (found in the lowest levels of evidence, see Table 3) limits the conclusions that can be drawn from this scoping review and, even though we are aware of its relevance, we must take it with some caution.

It is for this reason that it is essential that, as a profession, we continue to develop a rigorous body of knowledge in pedagogical practices or learning contexts that contribute to the formation of professional identity in Occupational Therapy education related to the development of professional intelligence. We must be more rigorous in the designs of research, in the validated measurements of these practices or contexts, we even must be able to construct and describe a specific taxonomy of these practices.

In this scoping review, we illustrate pedagogical practices or learning contexts that contribute to the formation of professional identity in Occupational Therapy education related to the development of professional intelligence and results of the same published in research, in addition to providing a general mapping of the different experiences and their development in the profession.

### 4.4. Limitations

In order to gather the highest amount of data, searches were carried out in international databases in Spanish and English. Even if the selected coverage is considerable, it must be taken into account that articles not published in these languages have not been included. 

By limiting ourselves to a description of empirical intervention articles, we have not been able to get a full picture of the literature that exists (monographs, doctoral theses, conferences, communications, posters, or grey literature in scope).

On the other hand, we do not know the effectiveness of the pedagogical practices, contexts and experiences described in the results, since the scientific quality of the articles was not evaluated in this scoping review.

### 4.5. Recommendations for Future Research 

It is necessary to review the educational curricula to detect how the pedagogical practices that facilitate the understanding of professional identity and professional intelligence are implemented and to detect if these programmes are adjusted to the studied reality or if they are tangential to it.

In addition, we believe it is necessary to analyse how professionals define their role, and if the teaching received was sufficient to build their identity or if, on the contrary, other factors appear that help this establishment. Moreover, future research should focus on examining the pedagogical practices of faculty, both role-emerging and fieldwork-oriented. By investigating fieldwork practices, further insight can be gained into the professional skills utilized by instructors and educators to cultivate professional acumen during occupational therapy academic training. In the meantime, research on role-emerging pedagogical practices will explore professional skills applied to identify clinical problems and intervention projects linked to the clinical practice. 

We are aware that it is important to contrast the results obtained with another research technique, so we suggest carrying out field research with students and professionals to analyse the repercussions of these different pedagogical practices. 

## 5. Conclusions

Given the purpose of this research we believe we can add that this scoping review revealed (a) the predominance of research on professional identity and development of professional intelligence, in the USA and UK, i.e., in an English-speaking culture, with a predominance of university-led research; (b) pedagogical practices for the forging of professional identity and the development of multifaceted professional intelligence, a range of contexts in which occupational therapy students learn, as well as the breadth of pedagogical practices and learning outcomes that can guide the design of occupational therapy professional identity training and development courses; (c) that professional skills, together with personal attributes and values, are at the core of the construction of professional identity and the development of multifaceted professional intelligence. This is coupled with an understanding of the professional role and a sense of belonging to a discipline.

## Figures and Tables

**Table 1:** Variables included in data charting.

Variable	Description
Article name	Name of the article
Year	The year of publication of the article has been detailed.
Authors	List of authors
Country	The country where interventions were carried out was collected.
Purpose/aim	Purpose/objective of the article as described in the text
Methods	Description of methodology, recruitment, analysis
Article type	Empirical (perspective and intervention), Review, Theoretical
Participant population	Undergraduates, Master, and Bachelor students and doctoral level.
Participant details	More details related to their learning stage
Terminology	Relevant terminology and definitions used
Notes	Notes for Analysts
Professional identity	Attitudes, values, knowledge, beliefs and skills shared with others within a professional group. Professional identity in this article refers to the behaviors and attitudes of professionals, perceptions and values, professional competencies and skills, job satisfaction, that is, these are categories that are not focused on training.
Professional intelligence	Skills needed for a successful career. In this article, within this variable we studied skills such as decision making, conflict resolution, time management, strategic planning, effective communication and the ability to lead and work in teams.
Learning context	Context of the pedagogical practice. It refers to the set of primary conditions that are necessary to construct knowledge about something (Figueiredo and Afonso 2005). The learning context described in the article includes the relevant circumstances where a person learns such as text, multimedia materials, the spoken word of the teacher.
PIFS	Other terms on the concept under study. PIFS or Professional Identity Formation (PIF) which refers to the process of internalising the norms, values and standards of behaviour of a professional group so that each person comes to think, act and feel like a member of that community (Janke et al. 2021). PIFS refers to the specific training to obtain professional identity, and to the categories used in theoretical training models. Important events and transitions (Schlossberg model) were included, terms such as reflection, dialogue and conscious decision making (Baxter Magolda Model).
PIFLO	Professional identity formation learning outcomes. It is the professional identity formation learning outcome associated with pedagogical practice (Simmonds et al. 2020). PIFLO included the internalization of the norms and values of a professional group, the adoption of behaviors and professional practices, the development of professional skills and the identification with a professional community.
Pedagogical practice	The pedagogical practice described in the article. It refers to all the tools, strategies and activities that teachers use for their classroom work; these practices are influenced by educational models (Patiño 2006). The pedagogical practice described in the article included all the tools, strategies and activities used.

PIFS: Professional identity formation synonym; PIFLO: Professional identity learning outcome associated with the pedagogical practice.

**Table 2:** Learning educational contexts and pedagogical practice definitions.

**Fieldwork**	
	Through Fieldwork, students learn to apply the theoretical and scientific principles learned from their academic programmes and address the real needs of clients within the context of authentic practice settings (AOTA 2022). During Fieldwork experiences, each student develops competency in the occupational therapy process to determine clients’ occupational performance needs, identifies support or barriers affecting health and participation, and documents the interventions provided. Fieldwork also provides opportunities for the student to develop advocacy, leadership, interprofessional, and management skills in a variety of practice settings while incorporating principles of evidence-based practice and client-centered care. Finally, students develop a professional identity as an occupational therapist, aligning their professional judgments and decisions with the Standards of Practice and the occupational therapy code of ethics (AOTA 2022). The criteria established for a learning context to be considered Fieldwork are contexts where evidence-based practice principles are applied, environments where theoretical and scientific principles learned in academic programs can be implemented, but most importantly, where there is a supervisor who assists students in their learning. Learning contexts and pedagogical practices in Fieldwork are not “purist,” meaning that we can engage in reflective practice as long as it is conceptualized and limited as a practice of exploration and reflection.
**Reflexive practice**	
	Reflexive practice is a process (Benson and O’Reilly 2022), which enables a concomitant focus on reflexivity in action, organised around questions (Binyamin 2018). Reflexive practice is composed of three strategies: (a) positioning, which implies an awareness of our own relationships with occupational therapy interventions, with the people with whom we work, and with the production of knowledge in which we are engaged. That is, reflective practice concerns a sense of our positionality and positioning; (b) navigating, in which reflective practice in-volves navigating our way as the research proceeds and thereby actively learning from a reflexive approach; (c) interpreting, where the reflective practice informed by practice theory also recognizes reflexivity to understand how we intervene with the individuals and human groups with whom we carry out work. The criteria established for a practice to be reflective must be an intentional and systematic activity that involves the critical exploration of one’s own occupational therapy practice. Another criterion is that it must be oriented towards action and change, and finally, it is established that the reflection of that practice must involve the consideration of multiple perspectives. For a practice to be classified within this category, it must be a continuous and interactive process.
**Role-emerging placements (REP)**	
	Field placements undertaken by occupational therapy students are traditionally in established health care settings. However, it has been suggested that such settings may not adequately prepare students to work in the more diverse environments in which therapists increasingly find themselves. Changes in health and social care, with more emphasis on health promotion, wellbeing and community practice, mean that occupational therapy students must develop the knowledge, skills, and confidence to work in these key areas to remain viable as future practitioners. In response to this situation, pedagogical practices other than fieldworks have been developed, namely role-emerging placements (Clarke et al. 2014), which occur in a setting where there is no established occupational therapist role. Students in these placements are supervised daily by an employee within that setting and supported by an occupational therapist off-site. The criteria established to be classified within Role-emerging placements are the practices where occupational therapy students are in environments where there is an absence of occupational therapists. Therefore, as mentioned earlier, the main difference with Fieldwork is that there is no occupational therapy supervisor. There is an academic supervisor, but not a practice supervisor.
**Simulation**	
	Simulation is a pedagogical practice that recreates a situation in a scenario created to allow people to experience the representation of a real event for the purpose of practicing, learning, evaluating, testing or acquiring knowledge of human systems or performances (Vásquez and Hernándes 2021). Within this practice two types can be differentiated (Grant et al. 2021): (a) Interactive simulation in which this simulation takes the form of a patient or client represented by a suitably trained individual often following a standardised script or protocol; (b) Non-interactive simulation in which paper-based case studies are used, in which students read about a person’s needs and challenges, and video-based case studies, in which students can observe individuals either talking about, or performing, occupations they wish to develop. The criteria that we have established for a simulation context refer to situations where scenarios of real occupational therapy practice are imitated, environments where students can practice clinical skills in a safe setting, structured and planned activities with clear and specific objectives, integrated into a curriculum, and with evaluation.
**Other**	
	This section includes all those pedagogical practices which, due to their singularities, cannot be included in the previous ones and which cannot be a category of their own because they do not have homogeneous characteristics. The criteria established here cannot be part of the other categories.

**Table 3:** Learning educational contexts and pedagogical practice descriptions for intervention studies, *n* = 31.

			Pedagogical Practice Description		JBIe	
				RSM	EF	MF
**Fieldwork (*n =* 9)**						
	Toal-Sullivan (2006)		General study courses and discipline-specific practical training with 1000 h of fieldwork experience, useful for role adjustment, developing professional skills and facilitating understanding of professional identity in final year students. Fieldwork is discussed in the article as opportunities for practical learning in real clinical practice environments such as supervised clinical practices in settings like hospitals, rehabilitation centers, and community clinics.	QL		3
	Davis (2006)		Students undertook fieldwork in the community. A case study strategy was chosen to investigate students’ case management skills and develop professional identity. This article discusses communities of practice for identity development, where in Fieldwork, the group of people involved share common interests and objectives in an area.	QL		3
	Matsuda and Miller (2007)		During three consecutive fall semesters, fieldwork, role-play on team communication, theoretical understanding of cultural behaviour, health beliefs and practices, interviewing and active listening were designed in order to build professional skills for master students. The pedagogical practice used corresponds to Fieldwork where there is experiential learning based on field practice, critical reflection, and group discussion, which is commonly known as a competency-based approach.	QN	4.d	
	Humbert et al. (2012)		First to fourth-year students went through supervised fieldwork and service learning experiences during seventeen weeks (throughout the four years of training) and subsequently completed assigned reflective work on these experiences. The students who participated in the study had the opportunity to engage in a transcultural experience in a foreign country, which allowed them to have a field experience to apply theoretical and scientific principles.	QL		3
	Rodger et al. (2014)		Fieldwork with full-time tutor supervision. Students at these universities undertake full time placements lasting between 5 and 14 weeks over two semesters during their third and fourth undergraduate years, or second year for masters’ entry programs.	QL		3
	Ashby et al. (2016)		Students on a fieldwork programme (final year undergraduate) with the supervision of a tutor. It studies the perception of professional identity and how it is formed throughout one’s academic and clinical training.	QN	4.d	
	Hills et al. (2016)		Two or more eight-week blocks of educational fieldwork for third and fourth-year students.	MX	4.d	3
	Brown et al. (2019)		Students: (a) four-year bachelor of occupational therapy and (b) second-year master, completed between four and nine weeks of an educational practice with supervision by a tutor.	QN	4.d	
	Tal-Saban and Weintraub (2019)		An eight-month supervised community and classroom-based mentoring (CAST) programme for first-year students who participated in organisations with a service-learning education (SLE) vision where community service is integrated with instruction and reflection.	QN	3.e	
**Reflexive practice (*n =* 8)**						
	Greene (1997)		Undergraduates made six visits (one per week), each of which lasted one hour with the same client, followed by reflections and comments on the activity. Reflexive practice is observed as the author uses learning as an opportunity for occupational therapy students to reflect on their ethical decision-making process. Additionally, the author employs reflexive practice for students to develop critical thinking skills about complex ethical issues through in-class activities and discussions. The author emphasizes the importance of reflection after the learning experience.	QN	4.d	
	Stern (1997)		Course for second-year professional master’s students (two hours once a week for seven weeks), to integrate synthesis abilities, PBL skills, teamwork and professional reasoning. The article’s focus is on exploring the perceptions of occupational therapy students regarding a problem-based learning course. The study aimed to evaluate the students’ perceived benefits and drawbacks of problem-based learning, as well as their opinions on the adequacy of their own performance in the course.	QL		3
	Hammel et al. (1999)		Discussion groups (initial three-month course for first and second-year undergraduates) to seek information and formulate hypotheses about cases. The learner-centered approach was used to anchor content and knowledge and foster professional reasoning. Reflexive practice is seen as previously mentioned in problem-based learning programs. This article refers to the importance of teamwork as an activity for problem-solving, the value of critical reflection and self-evaluation for skills development.	QL		3
	Knecht-Sabres (2013)		Students in the last term of their didactic education in a master’s programme, made four visits during a three to a five-week period (each visit lasting between one and four hours) with older adults (>65) for evaluating and implementing occupational therapy services. Reflexive practice is used for the development of decision-making skills. In this study, reflexive practice is enhanced after practical experience. Students describe, through reflexive practice activities, the areas where they need to improve and develop an action plan to continue their learning.	MX	4.d	3
	Whitcombe (2013)		Course for students in their final year of an undergraduate programme, to integrate theoretical knowledge and experiences. Problem-based scenarios (case studies). Reflexive practice is focused on the problem-based learning course that students undertake. This type of learning, which is part of reflective practice, shapes students’ perceptions of their roles.	QL		3
	Barnard-Ashton et al. (2017)		Blended learning of students from the first to the fourth academic year. In a year of their training, they went through activities of analysis, reflection, critical reading and the use of new technologies. Reflexive practice is observed in this article when technology is integrated into the curriculum to improve the teaching-learning process.	QL		3
	Binyamin (2018)		A “Collaborative reflection” course of an eight-week duration to achieve: (1) reflective writing, (2) collaborative reflection, (3) discussion of ideas and different perspectives in undergraduates. Reflexive practice is observed in the development of professional identity through collaborative reflection on relational dilemmas. Reflexive practice is carried out in groups on dilemmas experienced during fieldwork.	QL		3
	Kreider et al. (2021)		During four semesters and intended for undergraduates, individual tutorials and monthly group meetings were held with discussions focused on knowledge sharing and reflective prompts. Reflexive practice is observed in this article when technology is integrated into the curriculum to enhance the teaching and learning process.	QL		3
**Role-emerging placements (REP) (*n =* 5)**						
	Knightbridge (2014)		Third and fourth-year undergraduates completed 300 h in REP. Working in pairs over two semesters, they researched and identified problems, formulated goals and objectives, developed an intervention plan, and finally, implemented and evaluated it.	QN	4.d	
	Clarke et al. (2014)		MSc pre-registration occupational therapy students were in REP for homeless people, refugees, children, adults and a drug-alcohol rehabilitation team. The REP were catalysts for understanding themselves, developing ways of being and representing occupational therapy practice.	QL		3
	Fitzgerald et al. (2017)		Third-year undergraduates spent five weeks in REP of low-income families, seniors, single parents, and people with disabilities.	QL		3
	Thew et al. (2018)		REP experience with occupation-focused practice in the Master’s level pre-registration occupational therapy programme, where they built self-confidence and professionalism.	QL		3
	Golos and Tekuzener (2019)		Students (second, third and fourth-year) participated in three role-based or role-emergent practice placements. They completed 1000 clinical hours to develop clinical and pragmatic reasoning skills in interventions.	QN	4.d	
**Simulation (*n* = 4)**						
	**Interactive**					
		Haracz et al. (2015)	A simulation was carried out in a course of occupational therapy in mental health (second year of bachelor programme) in two blocks of four hours. It was structured around case studies of occupational therapy.	QL		3
		Imms et al. (2018)	During five days (40 h), students (second year of undergraduate studies and first year of master studies) performed simulation activities to complete a professional task of field practice.	QN	1.c	
		Walls et al. (2019)	Over two days (80 min) with master’s students, multiple standard patient scenarios were simulated, and they were instructed to complete interviews, assessments, and interventions.	QN	4.d	
	**Not interactive**					
		Neistadt (1987)	First-year master’s students carried out a simulation process with clinical history review, assessment, hypothesis elaboration, use of reasoning (procedural and interactive) and intervention planning to establish priorities (ethical reasoning).	MX	4.d	3
**Other (*n =* 5)**						
	Botkin (1979)		Senior students attended a Speaking Group, (1 h per week for 2 months), to come to a shared understanding of the importance of participation, feedback, goal setting, common role awareness and the decision-making process.	EO		5
	Thompson and MacNeil (2006)		Three-month seminar for postgraduate students, instructional methods combined classroom discourse and learning activities.	QL		3
	Parra-Esquivel et al. (2017)		Each group was assigned a key topic for the development of professional identity and with the help of IT, learning in the educational field was enhanced.	QL		3
	Psychouli et al. (2020)		An online collaboration experience between ten groups of five master’s and bachelor’s students from Cyprus and USA, carried out an exposition after having selected a population and conducting readings and online searches to gather information.	MX	4.d	3
	Walsh and Pollard (2020)		Online module (12 weeks) of a master’s course to promote students’ political competence while in complex health and social care settings. Explored: power relations through critical theories, social constructionists, international politics, human rights law and concepts such as occupational justice.	QL		3

RSM: Research study method; JBIe: JBI level of evidence; EF: Effectiveness; MF: Meaningfulness; QL: Qualitative methods; QN: Quantitative methods; MX: Mixed; EO: Expert opinion.

## Data Availability

Not applicable.
